# Selection and validation of reference genes for normalization of qRT-PCR data to study the cannabinoid pathway genes in industrial hemp

**DOI:** 10.1371/journal.pone.0260660

**Published:** 2021-12-20

**Authors:** Michihito Deguchi, Shobha Potlakayala, Zachary Spuhler, Hannah George, Vijay Sheri, Ruba Agili, Aayushi Patel, Sairam Rudrabhatla

**Affiliations:** The Central Pennsylvania Research and Teaching Laboratory for Biofuels, Penn State Harrisburg, Middletown, Pennsylvania, United States of America; Louisiana State University College of Agriculture, UNITED STATES

## Abstract

There has been significant interest in researching the pharmaceutical applications of Industrial hemp since its legalization three years ago. The crop is mostly dioecious and known for its production of phytocannabinoids, flavonoids, and terpenes. Although many scientific reports have showed gene expression analysis of hemp through OMICs approaches, unreliable reference genes for normalization of qRT-PCR data make it difficult to validate the OMICs data. Four software packages: geNorm, NormFinder, BestKeeper, and RefFinder were used to evaluate the differential gene expression patterns of 13 candidate reference genes under osmotic, heavy metal, hormonal, and UV stresses. *EF-1α* ranked as the most stable reference gene across all stresses, *TUB* was the most stable under osmotic stress, and *TATA* was the most stable under both heavy metal stress and hormonal stimuli. The expression patterns of two cannabinoid pathway genes, *AAE1* and *CBDAS*, were used to validate the reliability of the selected reference genes. This work provides useful information for gene expression characterization in hemp and future research in the synthesis, transport, and accumulation of secondary metabolites.

## Introduction

Industrial hemp (*Cannabis sativa* L.) has a rich history in the civilization of humans because it can provide both phytochemicals and lignocellulosic biomass. This crop originated in Eurasia and is useful all over the world, largely as a fiber crop [1]. Following the emergence of more economically helpful fiber crops, the demand for hemp reduced, and the purpose shifted to usage as a food additive. Hemp seed contains essential fatty acids and proteins and gamma-linolenic acid, which has many health benefits [2]. Hemp seeds and oils are also used to produce nutritional supplements and cosmetics.

Recently, attention has been focused on its rich repertoire of pharmaceutical compounds [3]. Hemp produces a diverse array of phytocannabinoids, terpenes, and phenolic compounds with prominent nutraceutical potential [4]. Among them, phytocannabinoids are the most well-known phytochemicals. The predominant compound, cannabidiol (CBD) and tetrahydrocannabinol (THC) followed by cannabigerol (CBG) and cannabichromene (CBC) are highly promising compounds to improve the quality of human health. They act as therapeutic agents for central nervous system diseases such as epilepsy, inflammation, anxiety, and neurodegenerative disorders such as Parkinson’s, Huntington’s, Tourette’s syndrome, and Alzheimer’s [3]. Terpenes present an array of pharmacological properties, including anxiolytic, antibacterial, anti-inflammatory, and sedative effects on human diseases [5–7].

The increasing popularity of hemp-based phytochemicals has spurred the comprehensive analysis of genome and gene expression studies [8–11]. These analyses are necessary to identify the genes involved in secondary metabolite pathways [12, 13] and have helped to discover transcription factors which are the key proteins that positively or negatively control the synthesis of secondary metabolites [14, 15]. Accurate gene expression studies such as Northern blotting [16], ribonuclease protection assay (RPA) [17], serial analysis of gene expression (SAGE) [18], and quantitative real-time PCR (qRT-PCR) [19] are essential to confirm genomic and transcriptomic data. Among these methods, qRT-PCR is the most frequently used for gene expression analysis because of its high sensitivity, specificity, accuracy, reproductivity, and relatively low cost [20]. qRT-PCR also requires a minimal amount of RNA compared to hybridization-based methods.

The assessment of different samples in the same parameter is evaluated by qRT-PCR [21]. The analysis is used to detect changes in the expression of genes of interest relative to a reference gene. Because of the variances incurred during RNA extraction, DNase treatment, and cDNA synthesis, the reliability of gene expression results can be affected by sample size, RNA degradation, reverse transcription efficiency, and cDNA quality [22]. To provide accurate and reproducible results of gene expression profiles, researchers use reference genes as internal controls. Expression levels vary depending on different environmental conditions, making it critical to identify appropriate and reliable reference genes for each experimental set-up in the respective plant tissue and genotype to prevent biased or misinterpreted data [23–25].

Mangeot-Peter et al. (2014) [26] identified suitable reference genes in hemp stem tissue for accurate expression profiling of cell wall synthesizing genes. Subsequently, Guo et al. (2018) [27] studied seven reference genes in various hemp tissues, such as root, stem, leaf, and flower. To our knowledge, there has been no reports on the suitability of reference genes for normalization of gene expression in hemp under different experimental conditions. The *F-box* gene is used for hemp gene expression analysis. However, its stability under stress conditions has not been analyzed leading to inaccurate normalization of qRT-PCR analysis [26]. This study aims to evaluate stable reference genes under different abiotic stresses/hormone stimuli in hemp.

## Materials and methods

### Plant material, greenhouse conditions, generation of clones, growth, and care

Industrial hemp and medical marijuana plants share *Cannabis sativa* as their common scientific name. Therefore, in this paper, the authors referred to industrial hemp as “hemp”, to distinguish it from medical marijuana.

The hemp strain, Thunderbird, was grown following the approved guidelines for industrial hemp provided by the Pennsylvania Department of Agriculture—Bureau of Plant Industry under the regulated permits IH-16-P-2017 and IH-17-P-2017.

Greenhouse conditions were maintained at 25°C with a 14-hour light photoperiod at 25–40μEm^-2^s^-1^. Hemp clones were achieved by collecting a 3-inch segment containing two axillary buds and coating the 45-degree cut with Clonex Rooting gel (Hydrodynamics International, Inc. Lansing, MI). The explant was placed in Root Riot plugs (Hydrodynamics International, Inc. Lansing, MI) and maintained under propagation domes for two weeks at which point they were transferred to four-inch pots containing high porosity soil, HP Mycorrhizae from Pro-Mix (Rivière-du-Loop, Québec, Canada). Genetically identical clones of similar size were obtained by vegetative cuttings from the same female mother plant.

Clones were kept under 24-hour light under propagation domes and 12-hour light during the pre-flowering and flowering periods. The temperature was maintained at 25°C. The humidity for rooting clones was maintained at 65% and decreased gradually to 45% once the clones started to flower. Lost Coast Plant Therapy (Plant Protector, Inc. Loleta, CA) was applied to the clones biweekly at a dilution of 30mL per 4 liters to control pests.

### Plant stress treatments

All treatments except UV light treatment were performed in the greenhouse on the same day. Four-week-old, cloned plants grown in small pots were soaked in water including 100mM of mannitol (drought stress), 100mM of NaCl (salt stress), 200μM of CuSO_4_, 100μM CdCl_2_, or 100μM of Pb(NO_3_)_2_ and 200μM of ZnSO_4_ (heavy metal stresses), and100μM abscisic acid (ABA), 100μM of methyl jasmonate (MeJA), 1mM of gibberellic acid (GA_3_), or 100μM of salicylic acid (SA) (hormone treatments) for eight hours. For UV treatment, hemp cloned plants were exposed to UV-C radiation for 10 minutes. After each stress treatment, the 3^rd^ and 4^th^ leaves from the top of the plant were sampled and immediately frozen in liquid nitrogen and stored in the -80°C freezer until total RNA was extracted. All the treatments were performed in three biological replicates. For the mock plants, distilled water was used to soak the hemp plants.

### Total RNA extraction and cDNA synthesis

Total RNA was extracted from 100mg of each plant sample using the Spectrum^™^ Plant Total RNA kit (Sigma Aldrich, St. Louis, MO, USA). RNA concentration and absorbance ratios (A260/280 and A260/230) were measured using a NanoVue Plus spectrophotometer (General Electric Healthcare Limited, UK) to measure the quantity and quality of the total RNA. After treatment with DNase I (TaKaRa Bio, Dalian, China) to remove genomic DNA contamination, 2μg of total RNA was used to synthesize cDNA using the high-capacity cDNA reverse transcription kit (Applied Biosystems, Foster City, CA) according to the manufacturer’s protocol.

### Candidate reference genes selection, primer design, and PCR reaction

We identified 13 candidate reference genes (Table 1) and two target genes using a BLAST search from NCBI (https://www.ncbi.nlm.nih.gov/) and the *Cannabis sativa* genome browser gateway (http://genome.ccbr.utoronto.ca/cgi-bin/hgGateway). The *Cannabis sativa* genome browser gateway is based on the Purple Kush strain of medical marijuana. Primers were designed based on the sequences of 13 genes using Primer3 Plus (http://www.bioinformatics.nl/cgi-bin/primer3plus/primer3plus.cgi) with the criteria: amplicon size 80–200bp, primer size 18–24bp, Tm 60°C, GC content 45–60%. All primer sequences are listed in Table 1.

**Table 1:** Gene description, primer sequences, and PCR efficiency for the selection of hemp reference genes.

Gene name	Gene symbol	Accession number	Arabidopsis homolog (Homology)	Primer Sequence (5’ → 3’)	Amplicon Length (bp)	PCR Efficiency (%)	Regression Coefficient (R2)
18S ribosomal RNA	18S	XM_030651156.1	AT5G57280 (72.7%)	GAGAATGGGCATGAGTGGAT	140 bp	91.22	0.9956
				GCCCCATCAATAAGACCAGA			
40S ribosomal protein	40S	XM_030628282.1	AT3G52580 (82.7%)	TGCCACTGGTGGTAACAAAA	123 bp	98.44	0.9901
				CTGTCAGTTGGGATGGGAGT			
Chalcone synthase	CHAL	XM_030653640.1	AT5G13930 (72.0%)	GCCAGCCCAAATCAAAGATA	156 bp	95.15	0.9933
				CAGTTCCACCAGCAAAACAA			
E3 ubiquitin-protein ligase	UBE3	XM_030633681.1	AT1G79380 (73.2%)	GCTCCTTACCGTCAGACTCG	150 bp	105.44	0.9981
				GTTTGCGGCAGATAGGACAT			
Elongation factor 1- α	EF-1α	JP480592	AT1G09640 (74.0%)	GCCCTGTCTTTGAGAGCAAC	111bp	95.51	0.9991
				CAATCCACTGCTCAATGTGG			
F-box family	F-box	XM_030628913.1	AT5G06550 (73.8%)	GGGTCCAAGAAATGGGTTTT	81bp	111.85	0.9988
				TGCTACCTCAGCACCATCAG			
Glyceraldehyde-3- phosphate dehydrogenase	GAD	XM_030636658	AT1G42970 (78.4%)	TCCACTGACTTTGTGGGTGA	115bp	97.27	0.9983
				TGTAACCCCACTCGTTGTCA			
Phytochelatin synthase	PCS1	JP458288	AT5G44070 (70.7%)	TGAAGGTTTGGTGTGGTGAA	99 bp	94.10	0.9893
				TACACCGGTGAACCACTTGA			
Protein phosphatase 2A subunit	PP2A	XM_030625838	AT1G10430 (72.5%)	GCTTTGATACCCCTCCACAA	105 bp	100.98	0.9893
				AGTATCCGCGAGCTTGACAT			
Sand family	SAND	JP472489	AT2G28390 (80.4%)	GTTGCTGATTCCGGTGTTTT	95bp	113.87	0.9921
				TCATCTGGATGCAGTGAAGC			
TATA-box-binding protein	TATA	XM_030646209.1	AT1G55520 (80.3%)	TTTCCAGGCTTGATTTACCG	94 bp	107.17	0.9994
				CCCTCACCTTTGCTCCTGTA			
TIP41-like family protein	TIP41	JP466741	AT4G34270 (74.5%)	GCGACTGTGGAAATGGAAGT	162 bp	106.88	0.9985
				TTCTCCCCACTGTTCAAAGG			
Tubulin α -1	TUB	JP479709	AT1G64740 (79.9%)	CTCGGCTGAGAAAGCATACC	102 bp	105.77	0.9983
				CCATGCCTAGGGTCACACTT			
Acyl-activating enzyme 1	AAE1	JN717233		CGTTGCTTTTCCTCTTCTGG	106 bp	103.99	0.9997
				TTTCTGTGCCACCACACATT			
Cannabidiolic acid synthase	CBDAS	AB292682		GATCCGCTGGGCAGAACGGT	188 bp	111.01	0.9994
				CAGCAATTCCATTCCCTCAT			

### qRT-PCR amplification

PCR was performed using cDNA as the template to confirm the specificity of the primers to the target genes. Using a 2% (w/v) agarose gel, all PCR products were analyzed using electrophoresis to confirm a single band of the expected size for each of the primer pairs. To test the PCR amplification efficiency, the regression coefficient (R^2^) for each gene was calculated using a standard curve generated from a fivefold dilution series of cDNA (1, 1/10, 1/100, 1/1000, and 1/10000) for each primer pair. Based on the slopes of the standard curves, the PCR efficiency of each gene was determined from the respective logarithm of the cDNA dilution and plotted against the mean threshold cycle (Ct) values. The PCR efficiency was calculated using the equation:

E(%)=(10−(1/slope)−1)×100,

where E is the efficiency, and the slope is the gradient of the best-fit line in the linear regression. qRT-PCR was performed with 5μL of SYBR Select Master Mix (Applied Biosystems, Waltham, MA, USA) in a 10μL total reaction mixture containing 400nM of the gene-specific primers and 1μL of cDNA. PCR reaction was performed using a Bio-Rad CFX96 system (Bio-Rad, Hercules, CA, USA) under the following reaction conditions: Initial denaturation at 95°C for 10 minutes, 35 cycles of 95°C for 10 seconds, and 60°C for 1 minute. Three technical replicates were used for each biological replicate and average Ct was used for data analysis. As a negative control, water and total RNA were used instead of cDNA to confirm that there was no amplification from contaminated DNA or hemp genomic DNA.

### The stability of reference genes and statistical analysis

Boxplots of quantitative cycle (Cq) values for the 13 candidate reference genes were depicted in all leaf samples with every treatment using the boxplot R package to show the variation of each gene expression. The expression of 13 reference genes was analyzed under 11 different stresses using four algorithms, geNorm, NormFinder, BestKeeper, and RefFinder to rank the stability of the candidate reference genes. The pairwise variation (Vn/Vn+1) between two sequential normalization factors was calculated with geNorm to determine the optimal number of candidate reference genes for accurate normalization [28].

### Validation of identified reference genes

Two cannabinoid pathway genes, *CBDAS* and *AAE1*, were used as target genes to validate the reliability of the selected reference genes using the most stable candidate reference genes and the least stable reference genes. Primer design and calculation of PCR amplification efficiency for these genes was performed as described above. Relative gene expression levels of *CBDAS* and *AAE1* were calculated using the 2^−ΔΔCt^ method (28). Statistical analysis was performed using a paired t-test (a = 0.05) (28).

## Results

### PCR specificity and amplification efficiency of the candidate reference genes

**Fig 1:**
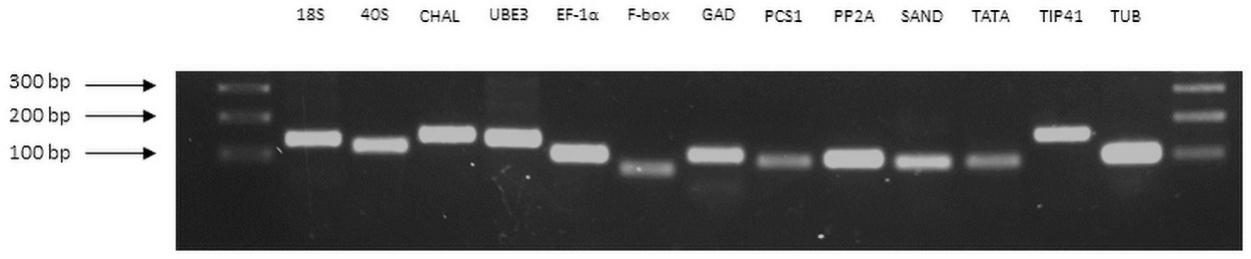
Amplification results for 13 candidate genes using cDNA synthesized from hemp leaf sample to confirm primer specificity and amplicon size.

Thirteen reference genes (*18S*, *40S*, *CHAL*, *UBE2*, *EF-1α*, *F-box*, *GAD*, *PCS1*, *PP2A*, *SAND*, *TATA*, *TIP41*, and *TUB*) were identified from NCBI and the *Cannabis sativa* genome browser gateway based on a homology search with *Arabidopsis* genes (Table 1). Primers were designed and used to confirm their specificities based on their amplification efficiency and specificity (Table 1). Single bands were amplified in agarose gel electrophoresis for all the gene primers with predicted sizes (Fig 1). For the qRT-PCR amplification, the PCR efficiency (%) ranged from 91.22 to 113.87, and the regression coefficient (R^2^) varied from 0.9893 to 0.9994 (Table 1).

### Ct values of candidate reference genes

**Fig 2:**
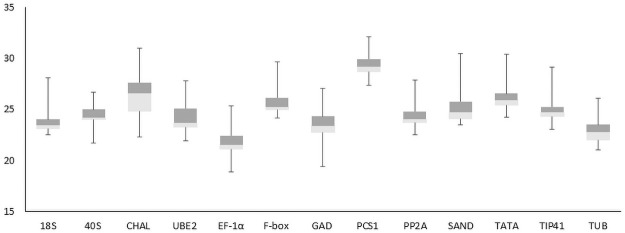
Expression level variability of each candidate reference gene to examine all leaf samples (n = 36). Boxes show the 25^th^ and 75^th^ percentiles, whisker caps represent the minimum and maximum values, lines across the box represent the median Ct-values.

Transcript abundances of 13 candidate reference genes were assessed by qRT-PCR for each gene, tested in triplicates across all 11 treatments and a control, which was 36 biological samples (Fig 2). A majority of the candidate reference genes Ct values ranged from 20 to 30. The lowest expression level with the highest Ct values between 27.4 and 32.1 was *PCS1*. The *EF-1α* gene showed the highest expression level, with the lowest Ct values ranging from 18.9 to 25.3. The *CHAL* gene displayed the highest difference among all 36 samples tested, with a minimum Ct value of 22.3 and a maximum Ct value of 30.9. These Ct value analyses showed that the transcription levels of candidate reference genes are unstable under different stress conditions.

### Analysis of reference genes by geNorm

The geNorm was used for evaluating the expression stability of the 13 candidate reference genes (Table 2). Data analysis was calculated based on individual 11 different treatments and three different groups of treatments such as osmotic stress (OS: mannitol, NaCl), heavy metal stress (HM: CdCl_2_, CuSO_4_, PbNO_3_, ZnSO_4_), and hormonal stimuli (PH: ABA, GA_3_, MeJA, SA). The total ranking was also shown by combining all 11 treatments together. This algorithm evaluated the stability of reference genes (M) based on the average pairwise variation of all tested genes [29]. In this analysis, the lower the M value, the more stable the gene expression. A reference gene that has an M value less than 1.5 is used for qRT-PCR. *PP2A* and *TIP41* were the most stable reference genes with the lowest M value (0.46) whereas *CHAL* had an M value of 1.07 and was ranked as the least stable gene. Individually, *EF-1a* and *SAND* were the most stably expressed genes under osmotic stresses with an M value of 0.22 while *F-box* and *TATA* were the least stably expressed genes. The *TUB* and *TATA* genes showed the lowest M values of 0.16 among all of the heavy metal stressed clones. Exposure to hormonal stimuli resulted in *PP2A* and *F-box* to be the most stable with an M value of 0.27 and *CHAL* to be the least stable with an M value of 0.75.

**Table 2:** Stability ranking of the 13 candidate reference genes in hemp leaf analyzed by the geNorm program under different stresses.

Rank	Total	OS	HM	PH	Mannitol	NaCl	CdCl2	CuSO4	PbNO3	ZnSO4	ABA	GA3	MeJA	SA	UV
1	PP2A	EF-1a	TUB	PP2A	EF-1a	EF-1a	TIP41	EF-1a	UBE2	18S	18S	TATA	18S	GAD	F-box
	0.46	0.22	0.16	0.27	0.21	0.12	0.06	0.06	0.06	0.00	0.06	0.00	0.00	0.10	0.06
2	TIP41	SAND	TATA	F-box	SAND	TIP41	TUB	PP2A	TIP41	40S	PCS1	TIP41	SAND	PCS1	GAD
	0.56	0.32	0.20	0.28	0.21	0.14	0.10	0.07	0.06	0.02	0.06	0.10	0.04	0.14	0.08
3	TUB	TUB	UBE2	TATA	TUB	SAND	18S	SAND	TATA	F-box	TATA	PP2A	F-box	F-box	TIP41
	0.58	0.32	0.24	0.29	0.31	0.18	0.11	0.09	0.24	0.04	0.06	0.14	0.08	0.16	0.12
4	EF-1a	PP2A	TIP41	TIP41	GAD	TUB	TATA	F-box	TUB	SAND	EF-1a	F-box	TATA	UBE2	PP2A
	0.62	0.64	0.25	0.35	0.46	0.23	0.12	0.11	0.25	0.08	0.07	0.17	0.28	0.19	0.16
5	SAND	TIP41	PP2A	SAND	PCS1	18S	F-box	TIP41	GAD	TATA	PP2A	18S	TIP41	EF-1a	EF-1a
	0.67	0.77	0.32	0.38	0.74	0.27	0.19	0.12	0.34	0.10	0.14	0.20	0.35	0.21	0.17
6	18S	18S	PCS1	TUB	40S	PP2A	EF-1a	UBE2	PP2A	PP2A	40S	TUB	PCS1	PP2A	UBE2
	0.72	0.91	0.36	0.42	0.92	0.32	0.21	0.15	0.43	0.12	0.16	0.25	0.39	0.23	0.20
7	UBE2	40S	SAND	EF-1a	TIP41	UBE2	GAD	TATA	PCS1	TUB	TIP41	SAND	PP2A	TATA	TATA
	0.75	0.97	0.41	0.46	1.00	0.40	0.22	0.17	0.53	0.16	0.17	0.28	0.42	0.27	0.22
8	PCS1	UBE2	EF-1a	UBE2	PP2A	40S	UBE2	PCS1	SAND	EF-1a	SAND	PCS1	40S	SAND	SAND
	0.79	1.00	0.47	0.49	1.07	0.43	0.24	0.20	0.61	0.18	0.19	0.31	0.46	0.32	0.24
9	GAD	PCS1	40S	PCS1	18S	CHAL	SAND	18S	40S	PCS1	F-box	EF-1a	TUB	TUB	40S
	0.82	1.05	0.53	0.51	1.16	0.47	0.27	0.23	0.76	0.22	0.21	0.34	0.49	0.36	0.26
10	40S	GAD	18S	18S	UBE2	PCS1	40S	TUB	EF-1a	UBE2	UBE2	UBE2	EF-1a	TIP41	18S
	0.85	1.10	0.58	0.53	1.23	0.55	0.31	0.27	0.86	0.25	0.23	0.38	0.56	0.40	0.30
11	F-box	CHAL	GAD	40S	CHAL	TATA	PCS1	CHAL	18S	TIP41	CHAL	40S	UBE2	18S	TUB
	0.90	1.20	0.63	0.58	1.43	0.60	0.36	0.32	0.94	0.27	0.25	0.43	0.61	0.51	0.35
12	TATA	F-box	F-box	GAD	TATA	F-box	PP2A	GAD	F-box	GAD	TUB	CHAL	GAD	40S	PCS1
	0.93	1.32	0.69	0.62	1.62	0.65	0.40	0.40	1.06	0.30	0.28	0.46	0.77	0.59	0.42
13	CHAL	TATA	CHAL	CHAL	F-box	GAD	CHAL	40S	CHAL	CHAL	GAD	GAD	CHAL	CHAL	CHAL
	1.07	1.41	0.87	0.75	1.73	0.69	0.53	0.49	1.39	0.58	0.30	0.48	0.93	0.77	0.59

*F-box* was ranked as the second least stably expressed gene under both osmotic and heavy metal stresses, but it was ranked as the first and second most stable gene under UV and plant hormone treatments, respectively. The *TATA* gene was least stably expressed under osmotic stresses but was among the top two and three under heavy metal stress and plant hormone stimulus, respectively. *CHAL* was the least stably expressed in response to UV light application.

**Fig 3:**
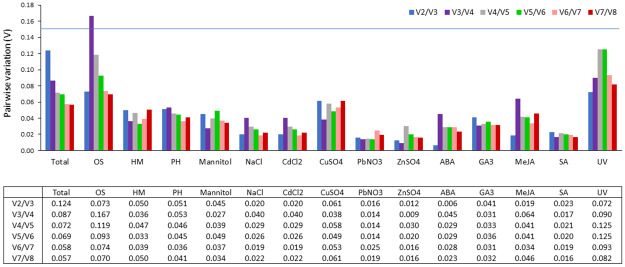
Determination of best reference gene number calculated by geNorm pairwise variation (Vn/Vn+1) under different stress treatments in hemp leaf.

geNorm can determine the minimal number of reference genes that should be used to obtain an accurate normalization. The optimal number of reference genes was determined based on the pairwise variation (Vn) between two normalization factors (NFn) composed of an increasing number of reference genes [29]. The threshold value (Vn/Vn+1 = 0.15) indicates if the number of reference genes less than or equal to the value of n is sufficient to use as a reference gene. As shown in Fig 3, the pairwise variation value V2/V3 of all experimental samples was less than 0.15, demonstrating that two reference genes should be sufficient for normalization under all conditions tested.

### Analysis of reference genes by NormFinder

NormFinder is a quantity-model-based software and uses complex statistical models to compute the variation between the expression of genes across different biological groups [30]. The lowest expression stability value represents the most stable reference genes. Results from the NormFinder analysis are summarized in Table 3.

**Table 3:** Stability ranking of the 13 candidate reference genes in hemp leaf analyzed by the NormFinder program under different stresses.

Rank	Total	OS	HM	PH	Mannitol	NaCl	CdCl2	CuSO4	PbNO3	ZnSO4	ABA	GA3	MeJA	SA	UV
1	EF-1a	TUB	PCS1	TATA	EF-1a	40S	TATA	SAND	SAND	EF-1a	TATA	TUB	PCS1	F-box	GAD
	0.355	0.038	0.237	0.161	0.104	0.143	0.029	0.029	0.154	0.076	0.029	0.161	0.076	0.05	0.029
2	TUB	SAND	EF-1a	TIP41	TUB	18S	18S	PP2A	PCS1	UBE2	PCS1	SAND	TIP41	EF-1a	F-box
	0.392	0.484	0.284	0.266	0.132	0.156	0.029	0.029	0.18	0.087	0.072	0.176	0.076	0.087	0.029
3	PCS1	EF-1a	PP2A	F-box	SAND	UBE2	TUB	EF-1a	PP2A	TIP41	18S	TATA	TATA	GAD	PP2A
	0.563	0.527	0.334	0.274	0.24	0.156	0.029	0.029	0.345	0.087	0.102	0.183	0.076	0.09	0.029
4	PP2A	GAD	SAND	PP2A	GAD	PP2A	TIP41	PCS1	40S	TATA	EF-1a	TIP41	40S	UBE2	TIP41
	0.616	0.701	0.334	0.357	0.755	0.236	0.074	0.119	0.396	0.122	0.144	0.183	0.29	0.116	0.058
5	TIP41	40S	UBE2	PCS1	PCS1	TUB	40S	TIP41	EF-1a	PCS1	40S	EF-1a	TUB	PCS1	EF-1a
	0.63	0.789	0.427	0.383	0.758	0.403	0.239	0.134	0.631	0.133	0.149	0.233	0.404	0.181	0.115
6	SAND	PCS1	TIP41	EF-1a	TIP41	TIP41	UBE2	F-box	18S	TUB	PP2A	PCS1	F-box	TATA	TATA
	0.673	0.889	0.436	0.385	1.115	0.511	0.246	0.134	0.768	0.169	0.149	0.253	0.472	0.246	0.165
7	18S	18S	TATA	TUB	40S	SAND	EF-1a	18S	UBE2	SAND	TIP41	PP2A	SAND	TUB	UBE2
	0.689	1.08	0.437	0.387	1.138	0.596	0.292	0.149	0.94	0.215	0.178	0.403	0.609	0.33	0.183
8	40S	PP2A	TUB	SAND	PP2A	PCS1	F-box	UBE2	TATA	18S	F-box	F-box	18S	PP2A	SAND
	0.736	1.122	0.511	0.418	1.174	0.596	0.292	0.277	0.947	0.323	0.223	0.418	0.609	0.358	0.239
9	UBE2	UBE2	40S	UBE2	18S	EF-1a	GAD	TATA	TIP41	40S	SAND	UBE2	EF-1a	SAND	40S
	0.752	1.179	0.535	0.449	1.716	0.641	0.35	0.303	0.97	0.323	0.228	0.475	0.62	0.4	0.255
10	GAD	TIP41	18S	18S	UBE2	TATA	PCS1	TUB	TUB	PP2A	TUB	CHAL	PP2A	TIP41	18S
	0.756	1.241	0.618	0.541	1.873	0.694	0.51	0.477	1.075	0.331	0.331	0.483	0.65	0.547	0.443
11	F-box	CHAL	F-box	40S	CHAL	F-box	PP2A	CHAL	F-box	GAD	CHAL	40S	UBE2	18S	TUB
	0.816	1.337	0.811	0.712	2.002	0.694	0.541	0.662	1.366	0.349	0.334	0.495	0.697	0.983	0.602
12	TATA	F-box	GAD	GAD	TATA	CHAL	SAND	GAD	GAD	F-box	UBE2	GAD	GAD	40S	PCS1
	0.907	1.606	0.889	0.795	2.152	0.705	0.544	0.711	1.467	0.394	0.368	0.508	1.654	1.028	0.802
13	CHAL	TATA	CHAL	CHAL	F-box	GAD	CHAL	40S	CHAL	CHAL	GAD	18S	CHAL	CHAL	CHAL
	1.711	1.717	1.79	1.346	2.203	0.847	1.234	0.982	3.141	2.112	0.385	0.515	1.723	1.733	1.529

The EF-1α and TUB genes were the most stably expressed in all samples and were ranked as fourth and third by geNorm, respectively. The F-box, TATA, and CHAL were ranked as the three least stable genes both by NormFinder and geNorm. The TUB, PCS1, and TATA genes were the most stably expressed under osmotic stress, heavy metal stress, and plant hormone stimuli, respectively. Compared to geNorm, TUB, PCS1, and TATA were ranked as third, sixth, and third positions in each category, respectively. The least stably ex-pressed reference genes under osmotic stress (CHAL, F-box, TATA), heavy metal stress (CHAL, F-box, TATA), and plant hormone stimuli (40S, GAD, CHAL) had similar rankings when compared to geNorm rankings. The GAD and F-box genes were found to be the most stable reference genes under UV stress while PCS1 and CHAL were the least stable. A similar trend was observed in the geNorm analysis.

### Analysis of reference genes by BestKeeper

The BestKeeper program is an excel-based algorithm and uses standard deviation (SD) and coefficient of variation (CV) data of the average Ct values for specific treatments [31] (Table 4). Lower CV ± SD values represent higher stability. When using the BestKeeper algorithm, genes with an SD value > 1 are undesirable reference genes [25]. When all samples were taken into consideration, *TATA* (SD = 0.74), *40S* (SD = 0.79), *PCS1* (SD = 0.84), EF-1a (SD = 0.90), and TUB (SD = 0.99) were determined to be reliable reference genes. *TATA* showed the lowest SD among all 13 reference genes in all samples and the SD values were greater than 1 in osmotic stress (1.29) and mannitol (1.82). The 40S gene was ranked as the second most stable candidate in all samples tested, but the SD value of 40S under NaCl and *PbNO*_*3*_ stresses were 1.09 and 1.36, respectively. *PCS1* was ranked at the third position in all samples tested and SD values were below 1 in any individual treatment and the three treatment groups. The *CHAL* gene displayed the highest SD value with 1.95 in all samples displaying that this gene is not ideal for gene expression normalization. The *CHAL* gene exhibited an SD value less than 1 only under GA_3_ (0.71), ABA (0.27), CdCl_2_ (0.58), and CuSO_4_ (0.98) treatments.

**Table 4:** Stability ranking of the 13 candidate reference genes in hemp leaf analyzed by the BestKeeper program under different stresses. Data after gene symbols mean Std ± CV%.

Rank	Total	OS	HM	PH	Mannitol	NaCl	CdCl2	CuSO4	PbNO3	ZnSO4	ABA	GA3	MeJA	SA	UV
1	TATA	PCS1	TATA	18S	TUB	GAD	40S	40S	GAD	F-box	SAND	18S	18S	18S	TUB
	0.74±2.85	0.68±2.28	0.48±1.86	0.23±1.00	0.24±1.05	0.62±2.60	0.24±1.01	0.40±1.63	0.18±0.79	0.29±1.13	0.04±0.18	0.22±0.96	0.38±1.63	0.20±0.86	0.04±0.17
2	40S	GAD	UBE2	PP2A	EF-1a	TATA	TATA	GAD	TIP41	18S	EF-1a	F-box	SAND	40S	PCS1
	0.79±3.22	0.76±3.19	0.50±2.11	0.40±1.69	0.44±2.05	0.76±2.87	0.31±1.20	0.40±1.64	0.24±0.99	0.31±1.31	0.07±0.32	0.24±0.98	0.38±1.54	0.20±0.84	0.16±0.50
3	PCS1	40S	TIP41	F-box	PCS1	PCS1	18S	PCS1	UBE2	40S	PCS1	PP2A	F-box	TATA	UBE2
	0.84±2.88	0.83±3.31	0.53±2.10	0.43±1.70	0.47±1.54	0.80±2.70	0.36±1.52	0.49±1.66	0.27±1.14	0.31±1.27	0.09±0.31	0.29±1.21	0.47±1.87	0.64±2.49	0.27±0.97
4	PP2A	TUB	40S	TATA	TIP41	F-box	PCS1	SAND	TUB	SAND	18S	TATA	PP2A	PP2A	TATA
	0.90±3.67	0.87±3.68	0.56±2.28	0.47±1.82	0.56±2.30	0.82±3.15	0.38±1.30	0.51±2.01	0.33±1.47	0.38±1.50	0.11±0.48	0.40±1.57	0.47±1.96	0.67±2.77	0.29±1.06
5	TUB	PP2A	TUB	40S	40S	40S	TIP41	F-box	TATA	TATA	UBE2	TIP41	TATA	GAD	PP2A
	0.99±4.28	1.05±4.33	0.57±2.46	0.52±2.19	0.58±2.29	1.09±4.34	0.38±1.50	0.53±2.07	0.38±1.47	0.40±1.53	0.13±0.56	0.40±1.64	0.76±2.97	0.69±2.97	0.40±1.47
6	18S	EF-1a	PP2A	SAND	SAND	UBE2	TUB	18S	PP2A	EF-1a	TATA	TUB	TIP41	PCS1	EF-1a
	1.01±4.23	1.07±4.80	0.58±2.40	0.59±2.41	0.60±2.39	1.11±4.55	0.40±1.75	0.56±2.33	0.62±2.57	0.42±1.93	0.13±0.52	0.56±2.54	0.84±3.48	0.69±2.45	0.42±1.73
7	GAD	UBE2	PCS1	TIP41	PP2A	PP2A	UBE2	PP2A	PCS1	PP2A	F-box	SAND	PCS1	F-box	F-box
	1.03±4.37	1.10±4.54	0.65±2.24	0.65±2.65	0.71±3.05	1.29±5.12	0.44±1.89	0.56±2.26	0.82±2.87	0.42±1.75	0.20±0.80	0.60±2.49	0.87±3.02	0.82±3.25	0.44±1.59
8	F-box	18S	SAND	EF-1a	18S	18S	PP2A	EF-1a	SAND	UBE2	40S	PCS1	40S	UBE2	GAD
	1.04±4.04	1.12±4.66	0.67±2.68	0.69±3.27	0.87±3.64	1.38±5.66	0.49±1.99	0.58±2.58	0.87±3.54	0.44±1.89	0.22±0.93	0.69±2.42	0.96±4.13	0.89±3.74	0.44±1.69
9	TIP41	SAND	18S	PCS1	GAD	TUB	EF-1a	TIP41	40S	TIP41	PP2A	CHAL	TUB	EF-1a	TIP41
	1.04±4.11	1.12±4.39	0.69±2.91	0.71±2.49	0.89±3.79	1.49±6.24	0.51±2.33	0.62±2.44	1.36±5.54	0.53±2.12	0.22±1.00	0.71±3.00	1.02±4.54	0.89±4.10	0.49±1.72
10	EF-1a	TIP41	GAD	TUB	UBE2	CHAL	F-box	UBE2	EF-1a	TUB	TIP41	GAD	CHAL	SAND	40S
	1.06±4.84	1.14±4.55	0.77±3.30	0.80±3.60	1.09±4.52	1.60±5.69	0.51±2.01	0.71±2.95	1.47±6.70	0.53±2.31	0.24±1.00	0.71±3.00	1.09±4.26	1.09±4.40	0.51±1.98
11	UBE2	TATA	EF-1a	UBE2	CHAL	TIP41	GAD	TATA	18S	PCS1	CHAL	EF-1a	EF-1a	TUB	SAND
	1.07±4.45	1.29±4.78	0.80±3.65	0.83±3.46	1.58±5.66	1.60±6.11	0.56±2.35	0.73±2.78	1.58±6.48	0.60±2.07	0.27±1.06	0.73±3.44	1.31±6.28	1.09±4.83	0.56±1.88
12	SAND	F-box	F-box	GAD	TATA	SAND	CHAL	TUB	F-box	GAD	TUB	40S	UBE2	TIP41	18S
	1.24±4.89	1.34±5.09	0.83±3.21	0.89±3.89	1.82±6.61	1.64±6.32	0.58±2.12	0.87±3.70	1.98±7.51	0.71±3.09	0.36±1.61	0.84±3.47	1.36±5.66	1.24±5.01	0.62±2.29
13	CHAL	CHAL	CHAL	CHAL	F-box	EF-1a	SAND	CHAL	CHAL	CHAL	GAD	UBE2	GAD	CHAL	CHAL
	1.95±7.36	1.55±5.54	1.65±6.16	1.21±4.89	1.87±6.97	1.69±7.42	0.71±2.83	0.98±3.55	3.11±12.00	1.73±6.54	0.40±1.73	0.87±3.61	2.02±9.01	1.93±7.92	1.40±4.81

### Analysis of reference genes by RefFinder

RefFinder is a web-based tool for comprehensive analysis that integrates geNorm, NormFinder, Delta Ct, and BestKeeper approaches [32]. The reference genes were ranked from the most stable (M value is the lowest) to the least stable expression (M value is the highest) using RefFinder (Table 5). Among them, the most stable candidate was the EF-1α gene, followed by the TUB gene in all samples. The EF-1α and TUB genes were also ranked in third and first places under osmotic stress conditions, respectively. The TATA gene was most stably expressed under heavy metal and plant hormone treatments while this gene was the least stable under osmotic stress. The CHAL gene was ranked as the least stable gene in all samples tested. The GAD and CHAL genes were the most and least stably expressed genes respectively under UV application, which was the same findings as to the NormFinder software.

**Table 5:** Stability ranking of the 13 candidate reference genes in hemp leaf analyzed by the RefFinder program under different stresses.

Rank	Total	OS	HM	PH	Mannitol	NaCl	CdCl2	CuSO4	PbNO3	ZnSO4	ABA	GA3	MeJA	SA	UV
1	EF-1a	TUB	TATA	TATA	EF-1a	18S	18S	EF-1a	UBE2	18S	PCS1	TATA	TIP41	F-box	GAD
	2.51	1.86	2.14	1.86	1.41	2.99	2.06	2.21	3.20	2.99	1.86	2.21	2.78	2.14	2.51
2	TUB	SAND	PCS1	F-box	TUB	40S	TATA	PP2A	PP2A	TATA	TATA	TUB	18S	GAD	F-box
	2.78	2.45	2.55	2.06	1.57	2.99	2.38	2.30	3.22	3.16	2.06	2.45	2.83	2.59	2.55
3	PP2A	EF-1a	UBE2	PP2A	SAND	UBE2	TUB	SAND	TIP41	EF-1a	18S	TIP41	SAND	PCS1	PP2A
	2.83	2.71	3.50	2.38	2.71	4.41	2.45	2.45	3.35	3.46	2.45	2.78	3.15	3.50	2.78
4	TIP41	PCS1	PP2A	TIP41	PCS1	TIP41	TIP41	PCS1	SAND	40S	EF-1a	SAND	TATA	EF-1a	TIP41
	3.76	4.24	3.66	3.60	4.16	4.46	2.78	4.90	3.72	3.57	3.36	4.45	3.31	3.56	3.83
5	PCS1	GAD	TIP41	18S	GAD	PP2A	40S	F-box	PCS1	SAND	SAND	PP2A	PCS1	UBE2	EF-1a
	3.83	4.47	4.12	5.62	5.18	5.09	4.61	4.95	3.74	3.87	4.90	4.58	3.35	4.90	4.95
6	40S	40S	TUB	EF-1a	TIP41	TUB	UBE2	TIP41	GAD	F-box	40S	F-box	F-box	TATA	UBE2
	6.16	4.53	4.23	6.40	5.86	5.48	6.40	5.48	5.07	4.24	5.89	4.76	4.24	5.24	5.24
7	18S	PP2A	EF-1a	SAND	40S	EF-1a	EF-1a	GAD	TATA	TUB	PP2A	18S	40S	18S	TATA
	6.24	6.16	5.92	6.40	5.96	5.53	6.90	6.45	5.18	6.40	6.34	5.05	5.66	6.04	5.86
8	TATA	18S	SAND	TUB	PP2A	SAND	F-box	18S	40S	UBE2	TIP41	PCS1	TUB	PP2A	TUB
	6.45	6.96	6.05	7.09	7.74	6.48	7.27	7.42	6.00	6.48	7.65	6.62	6.71	6.05	6.04
9	SAND	UBE2	40S	PCS1	18S	PCS1	PCS1	UBE2	TUB	PP2A	F-box	EF-1a	PP2A	40S	PCS1
	7.09	7.97	7.35	7.54	8.74	6.82	8.34	7.61	6.16	7.36	8.21	7.38	7.09	7.67	7.67
10	UBE2	TIP41	18S	40S	UBE2	GAD	GAD	40S	EF-1a	TIP41	UBE2	UBE2	EF-1a	SAND	SAND
	8.63	8.41	9.74	9.03	10.00	6.85	8.63	8.14	7.95	7.38	9.21	10.13	9.97	8.71	8.66
11	GAD	CHAL	GAD	UBE2	CHAL	TATA	PP2A	TATA	18S	PCS1	CHAL	CHAL	UBE2	TUB	40S
	8.91	11.47	11.22	9.19	11.00	6.85	10.61	8.89	9.23	7.93	10.74	10.44	11.24	8.89	9.24
12	F-box	F-box	F-box	GAD	TATA	F-box	SAND	TUB	F-box	GAD	TUB	40S	CHAL	TIP41	18S
	10.46	12.00	11.49	12.00	12.00	8.92	10.89	10.47	11.74	11.74	11.22	11.49	12.17	10.47	10.47
13	CHAL	TATA	CHAL	CHAL	F-box	CHAL	CHAL	CHAL	CHAL	CHAL	GAD	GAD	GAD	CHAL	CHAL
	13.00	12.47	13.00	13.00	13.00	10.44	12.74	11.47	13.00	13.00	13.00	11.93	12.24	13.00	13.00

### Validation of selected reference genes

**Fig 4:**
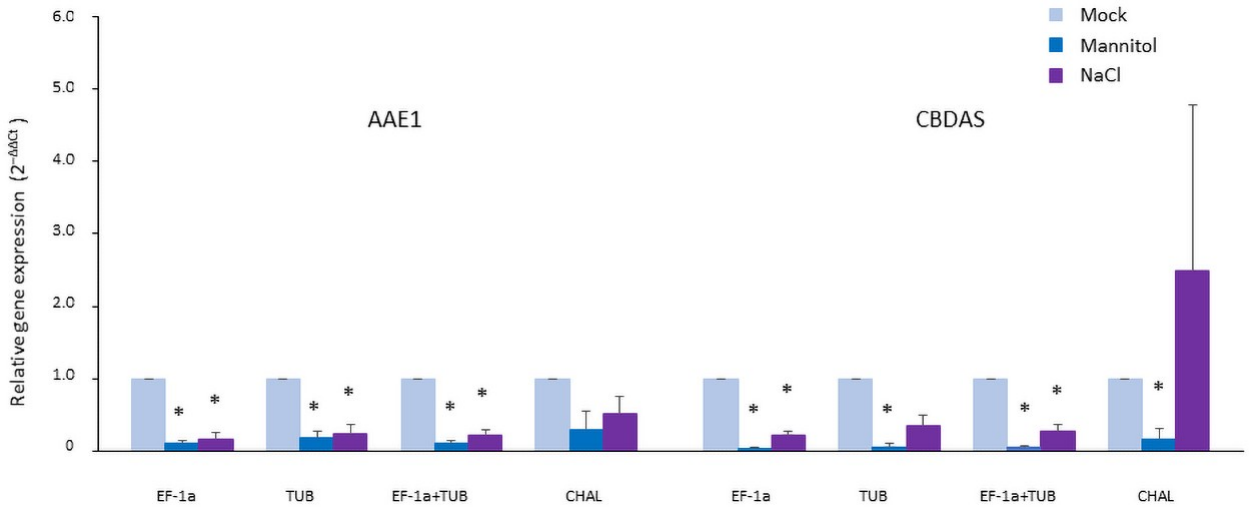
Relative expression of target genes in hemp leaf under osmotic stresses using most and least stably expressed reference genes for normalization. Error bars for qRT-PCR show the standard error of three replicates for *EF-1α*, *TUB*, and *CHAL*, and six replicates for a combination of *EF-1α* and *TUB*. The asterisk represents that there is a significant difference in the comparison with the mock treatment by the statistical analysis (P < 0.05) in paired T-tests.

To validate the selected reference genes, gene expression levels of AAE1 and CBDAS were measured (Fig 4). Each of the two most stable reference genes, EF-1α and TUB, a combination of these two stable reference genes (EF-1α+TUB), and the least stable reference gene (CHAL) were used as internal controls. AAE1 expression was significantly reduced under drought (Mannitol) and salinity (NaCl) stresses. EF-1α, TUB, and a combination of EF-1α and TUB were used for normalization of qRT-PCR analysis. There was no significant difference in the AAE1 expression between the mock treatment and osmotically stressed samples (Mannitol and NaCl) when CHAL was used as an internal control. The expression of CBDAS was also reduced under osmotic stresses when expression data was normalized with EF-1α, TUB, and a combination of EF-1α and TUB unless the CBDAS expression under NaCl stress was normalized with the TUB gene. When CHAL was used as a reference gene, CBDAS gene expression was reduced under mannitol treatment but there was no difference between mock and NaCl treatments.

## Discussion

Industrial hemp is from the plant species *Cannabis sativa* and has gained importance as a medicinal crop because of its potential to produce secondary metabolites such as cannabinoids, terpenes, and phenolic compounds [33]. According to Schluttenhofer and Yuan (2017) [4], hemp was cultivated for commercial or research purposes in at least 47 countries in 2017 and the global hemp market doubled from the year 2016 to 2020. Recently, a comprehensive gene expression analysis is aimed at elucidating the metabolic pathways for cannabinoids and terpene synthesis to improve hemp traits [34–36]. To validate this data, qRT-PCR analysis is suitable, however, appropriate hemp reference genes for accurate gene expression analysis have not been well established.

In this report, we evaluated 13 hemp reference genes under 11 different stress conditions. Research in other plant species has revealed that different environmental conditions would require unique reference genes to accurately interpret expression levels [37, 38]. Eleven different conditions including osmotic stresses, heavy metal stresses, plant hormone stimulus, and UV light application were reported to affect the cannabinoid synthesis [28, 39–41]. The results obtained from geNorm, NormFinder, BestKeeper, and RefFinder were not consistent, particularly BestKeeper which was much more distinct from the other software methods (Tables 2–5). This finding was expected because the BestKeeper algorithm evaluates data differently when compared to the three other programs [42].

To rank the most suitable reference genes across all treatments, there was no unanimity when compared to four different algorithms (Table 6), which represented the combined results obtained from four programs. In most cases, one candidate gene was ranked as the most stable gene by two or three programs, which showed that it might be a good reference gene under various treatments. Based on the combined rankings of the four programs used in our study, the overall results showed that the most stable genes varied while the least stable genes were almost the same. Across all plants tested, both NormFinder and RefFinder determined EF-1α as the most stable gene in all samples tested. In previous reports, EF-1α was demonstrated to be the most stable gene under different stresses in a variety of crops such as tobacco [43], maize [44], soybean [45], potato [46, 47]. Interestingly, this gene was not the most stable in any of the three groups (OS, HM, PH). The TUB gene appeared to be best the candidate under osmotic stresses because this gene was ranked as the most stable by both NormFinder and RefFinder which is consistent with the results obtained in Parsley under abiotic stresses [37]. Under heavy metal stress, *TATA* was ranked as the most stable gene by BestKeeper and RefFinder and the second most stable gene by geNorm. This gene was also ranked as the best reference gene in hormone stimuli by NormFinder and RefFinder. Interestingly, *TATA* was the least stable gene under osmotic stresses by geNorm, NormFinder, and RefFinder. *TUB* was the most stably expressed gene under osmotic stresses, whereas *TATA* was ranked as the best stable gene under both heavy metal stress and hormone stimuli. Unlike most stable genes, *CHAL* was found to be the least stable gene in most of the rankings with all samples and the three treatment groups (OS, HM, PH) when analyzed by all four programs. According to Wang et al. 2015 [48], candidate genes showing a high level of variation of Ct values should be avoided as internal controls. Our results showed that variation of the Ct value in *CHAL* was highest among all 13 reference genes (Fig 2), which is consistent with the fact that *CHAL* was ranked as the least stable by all four programs used in this study.

**Table 6:** Global ranking of the 13 candidate genes in hemp leaf analyzed by all programs: geNorm, NormFinder, BestKeeper, and RefFinder under different stresses.

Rank	Total	OS	HM	PH	Mannitol	NaCl	CdCl2	CuSO4	PbNO3	ZnSO4	ABA	GA3	MeJA	SA	UV
1	EF-1a	TUB	TATA	TATA	EF-1a	18S	TATA	SAND	UBE2	18S	PCS1	TATA	18S	GAD	F-box
2	TUB	EF-1a	UBE2	F-box	TUB	40S	18S	EF-1a	TIP41	TATA	18S	TIP41	SAND	F-box	GAD
3	PP2A	SAND	PCS1	PP2A	SAND	UBE2	TUB	PP2A	PP2A	40S	TATA	TUB	TIP41	PCS1	PP2A
4	PCS1	PCS1	PP2A	TIP41	PCS1	PP2A	TIP41	PCS1	PCS1	EF-1a	EF-1a	PP2A	TATA	EF-1a	TIP41
5	TIP41	40S	TIP41	SAND	GAD	TIP41	40S	F-box	SAND	SAND	SAND	F-box	F-box	UBE2	EF-1a
6	18S	GAD	TUB	18S	TIP41	TUB	UBE2	TIP41	TATA	F-box	40S	SAND	PCS1	TATA	UBE2
7	40S	PP2A	SAND	EF-1a	40S	PCS1	EF-1a	18S	GAD	UBE2	PP2A	18S	40S	PP2A	TATA
8	SAND	18S	EF-1a	TUB	PP2A	SAND	F-box	UBE2	TUB	TUB	TIP41	PCS1	PP2A	18S	TUB
9	TATA	UBE2	40S	PCS1	18S	EF-1a	PCS1	GAD	40S	PP2A	F-box	EF-1a	TUB	40S	PCS1
10	UBE2	TIP41	18S	40S	UBE2	TATA	GAD	40S	EF-1a	TIP41	UBE2	UBE2	EF-1a	SAND	SAND
11	GAD	CHAL	GAD	UBE2	CHAL	GAD	PP2A	TATA	18S	PCS1	CHAL	CHAL	UBE2	TUB	40S
12	F-box	F-box	F-box	GAD	TATA	F-box	SAND	TUB	F-box	GAD	TUB	40S	CHAL	TIP41	18S
13	CHAL	TATA	CHAL	CHAL	F-box	CHAL	CHAL	CHAL	CHAL	CHAL	GAD	GAD	GAD	CHAL	CHAL

In previous *Cannabis* qRT-PCR studies, the *F-box* gene has been used as an internal control for qRT-PCR [28, 49, 50]. Mangeot-Peter et al. (2016) [26] performed the reference gene analysis in hemp stems and concluded that the *F-box* gene was ranked as one of the most stable genes and *Histone 3* as the least stable gene among 12 reference genes tested under normal conditions. In this study, however, the *F-box* gene was the second least stable gene by RefFinder and the third least stable gene determined by both the geNorm and NormFinder programs when all samples were analyzed. Based on our group rankings (OS, HM, PH), *F-box* was ranked the second least stable genes by geNorm, BestKeeper, and NormFinder under both osmotic and heavy metal stresses. The *F-box* gene was stably expressed under normal conditions in hemp leaves (S2 Fig) and relatively stable under hormone stimuli as evident by its second position as ranked by both geNorm and RefFinder. These results show that *F-box* may not be a suitable reference gene for hemp qRT-PCR analysis under osmotic and heavy metal stresses. However, it could be acceptable as a reference gene under normal and plant hormone treatments. Overall, our study suggests that the *F-box* gene may not be the best reference gene for *C*. *sativa*, particularly in plant stress-related studies. Guo et al. (2018) [27] have studied the stability of reference genes in different hemp tissues/organs. They ranked ubiquitin and *EF-1α* as the most stable genes in leaf samples at different stages, and *PP2A* as the least stable gene in different organs. Notably, *EF-1α* was the most stable reference gene in our global ranking, showing that *EF-1α* is most stable under the normal condition and different abiotic stresses and hormonal stimuli.

Many studies have proved that the use of more than one reference gene enables the possibility of avoiding variations and achieving more accurate normalization of qPCR data [29]. To assess the optimal number of reference genes for the normalization of qRT-PCR data, we used the geNorm program to perform a stepwise calculation of the pairwise variation (Vn/Vn+1) between sequential normalization factors. In this analysis, a Vn/Vn+1 < 0.15 indicates that introducing an additional reference gene for normalization is unnecessary. Under all treatments, V2/V3 values were less than 0.15, which indicated that two reference genes were enough for the normalization of the real-time PCR data under any treatments in this study.

To validate the reliability of the selected reference genes, we measured the relative expression of two cannabinoids pathway genes using *EF-1α* and *TUB* as the most stable reference genes and *CHAL* as the least stable reference gene (Fig 4). Since CBDA content is decreased by the influence of osmotic stress [41], we measured the expression of *AAE1* and *CBDAS* genes that are involved in the rate-determining enzymatic reactions leading to CBDA synthesis under drought and salinity stresses [51, 52]. The expression of these two genes was significantly reduced under drought and salinity stresses when qRT-PCR data were normalized by *EF-1α*, *TUB*, and the combination of *EF-1α* and *TUB*. Notably, the expression level of both genes was normalized by *CHAL* under salinity stress and did not show a significant difference when compared with mock plants. These results suggest that *EF-1α* and *TUB* genes individually or in combination are suitable reference genes for hemp under osmotic stresses. Our validation study demonstrated the effectiveness of the ranking of reference genes by the programs used geNorm, NormFinder, and RefFinder.

To the best of our knowledge, this study is the first report that performed a systematic analysis of hemp reference genes under different abiotic stresses and hormonal stimuli. The knowledge obtained in this study could contribute to enhancing future hemp research related to the elucidation of mechanisms involved in the synthesis, transport, and accumulation of abundant secondary metabolites in hemp.

## Supporting information

S1 Fig Original gel picture for the amplification of 13 candidate genes using cDNA synthesized from hemp leaf sample to confirm primer specificity and amplicon size. (TIF)

S2 Fig Expression level variability of each candidate reference gene to examine among different tissues (n = 12). (TIF)

S3 Fig Hemp pictures before stress treatments. (PDF)

S4 Fig Hemp pictures after stress treatments. (PDF)

S1 Table Ct values to make a standard curve generated from different dilution series of cDNA for each primer pair. (XLSX)

S2 Table Ct values to study expression level variability of each candidate reference gene under different stresses in all leaf samples. (XLSX)

S3 Table Ct values to analyze the reference genes using four algorithms, geNorm, NormFinder, BestKeeper, and RefFinder. (XLSX)

S4 Table Ct values to evaluate the validation of identified reference genes. (XLSX)

S5 Table Ct values to study expression level variability of each candidate reference gene in different tissues. (XLSX)
