# Supplementary figures and images for: Selection and validation of reference genes for normalization of qRT-PCR data to study the cannabinoid pathway genes in industrial hemp
Source: PLoS One. 2021 Dec 20;16(12):e0260660. doi: 10.1371/journal.pone.0260660 (PMC8687539; doi:10.1371/journal.pone.0260660)

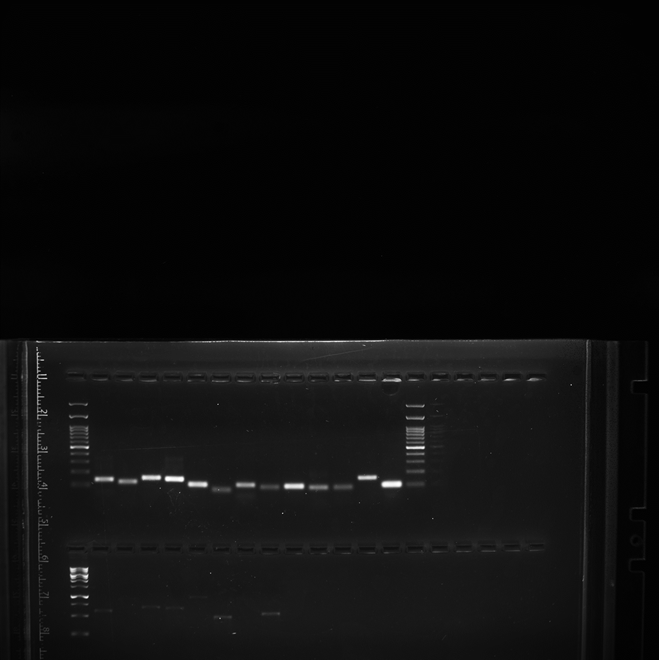

Supplement: S1 Fig — (TIF) [file pone.0260660.s001.tif]

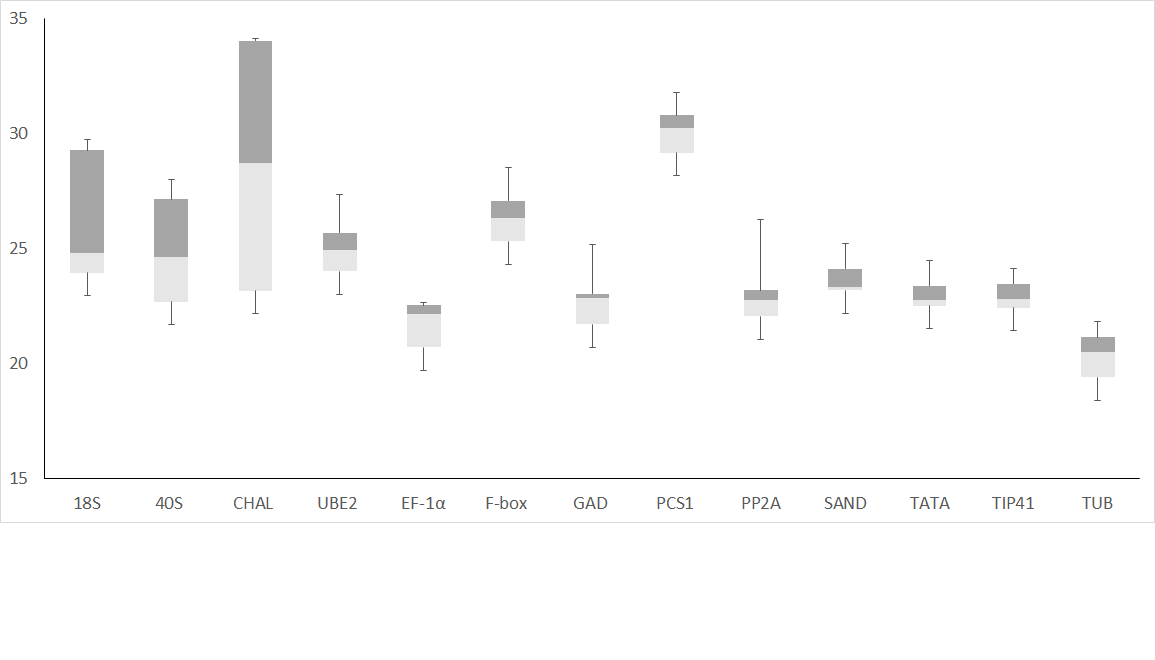

Supplement: S2 Fig — (TIF) [file pone.0260660.s002.tif]
