# Supplementary material for: Selection and validation of reference genes for normalization of qRT-PCR data to study the cannabinoid pathway genes in industrial hemp
Source: PLoS One. 2021 Dec 20;16(12):e0260660. doi: 10.1371/journal.pone.0260660 (PMC8687539; doi:10.1371/journal.pone.0260660)

Hemp pictures after stress treatments

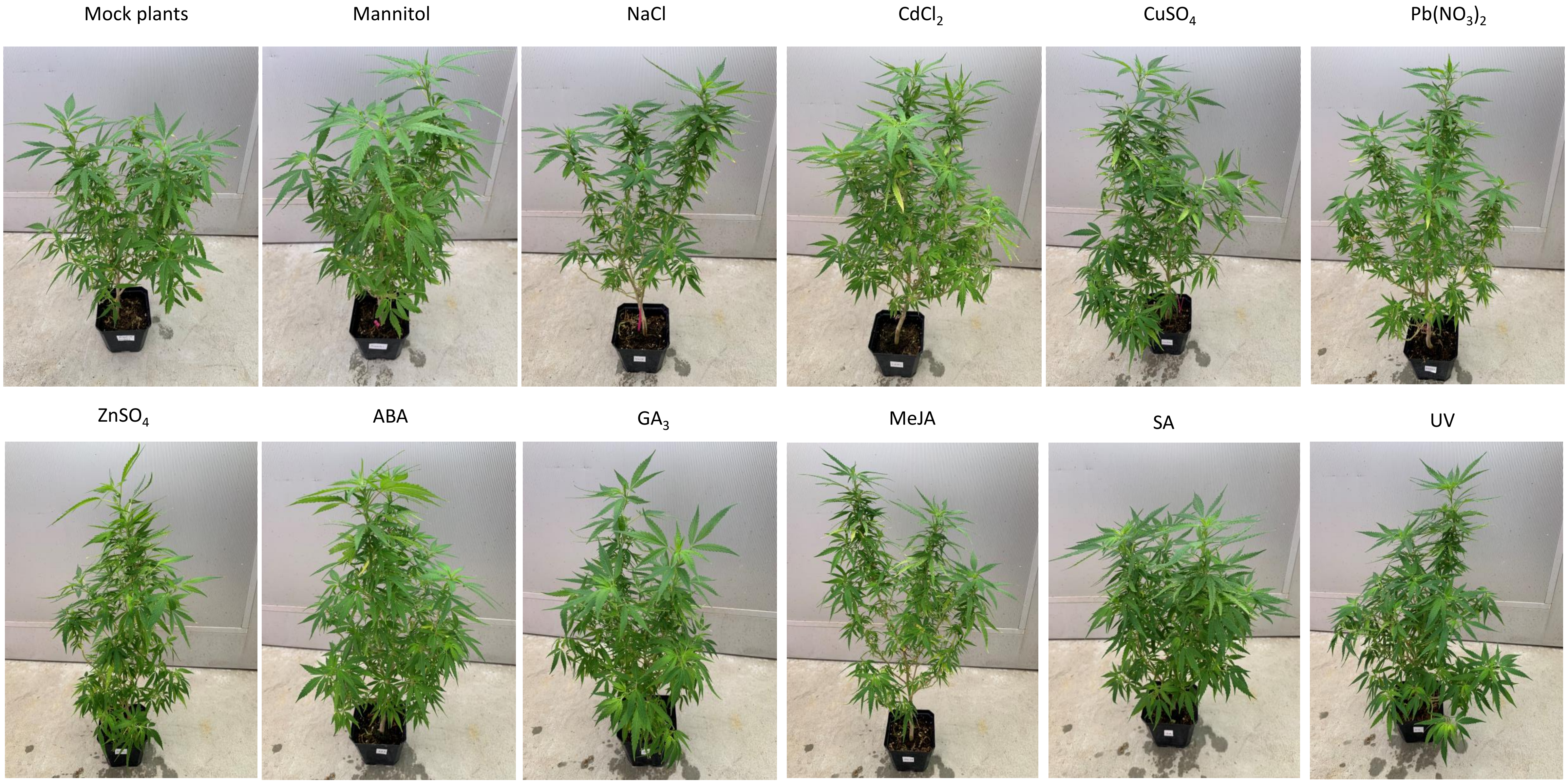

Supplement: S4 Fig — (PDF) [file pone.0260660.s004.pdf]
